# 

**Published:** 1890-05

**Authors:** 


					﻿ZMLA.Y, 18©O.
OLD SERIES
Vol XXV
NEW SERIES
Vol. VII.-No. 3.'
& 1855 v'
TOTirf
mEDIGAI^^DRk®
JOURNAL



W. F. WESTMORELAND, M.D.
H. V. M. MILLER, M.D., LL.D.
VIRGIL 0. HARDON, M.D.
F. W. McRAE, M.D,, M’n’g’ng Ed.
PUBLISHED BY JAMES P.HARRISON&.CQ,
iff.___________vjflTLANTA.GA. £&
SUBSCRIPTIONS S2.BO A YEAR.
				

